# The Family Check-Up Online: Investigating Patterns of Engagement in a Digital Intervention for Parenting Early Adolescents

**DOI:** 10.1007/s10935-025-00876-5

**Published:** 2025-10-18

**Authors:** Anna Cecilia McWhirter, Katherine A. Hails, Audrey C. B. Sileci, Anne Marie Mauricio, Elizabeth A. Stormshak

**Affiliations:** 1 Prevention Science Institute, University of Oregon, 1600 Millrace Dr, Eugene, OR 97405 USA; 2 College of Education, University of Oregon, Eugene, USA

**Keywords:** Family check-up, Parenting intervention, Digital health, Early adolescence

## Abstract

Early adolescence is a critical developmental period, and effective evidence-based parenting interventions during this time are crucial. Traditional parenting interventions are rife with barriers including cost, access, and availability of providers. The use of digital parenting interventions, such as the Family Check-Up (FCU) Online, can provide parents with needed supports and reduce barriers to access. The FCU Online is a flexible parenting intervention involving the development of parent training skills via an app and supplemental family coaching sessions. The intervention was adapted to respond to family-related stressors due to the COVID-19 pandemic in the Pacific Northwest of the United States. The current study investigated patterns of parent engagement in the FCU Online and their links to family contextual factors and outcomes, with the following questions: (1) What are the rates of parent intervention engagement in the FCU Online coaching and the app? (2) Did parent intervention engagement differ based on individual or contextual factors (e.g., depression, SES)? and (3) Does engagement level in the FCU Online predict differential outcomes for parents and their families six months after intervention enrollment? Two groups for coaching and two groups for the app were created based on *n* = 74 parents’ engagement (Typical or High Engagement). One-Way ANOVA’s and MANCOVA’s were used to address the second and third research questions, respectively. All parents engaged in the FCU Online intervention. Parents with higher depression and stress, lower parenting confidence, and who had adolescents with more problem behaviors were in the High Engagement group for coaching. No differences in app engagement were found based on contextual factors. Higher coaching engagement predicted improvements in quality time, and higher app engagement predicted reduced parent anxiety. The FCU Online may be successful in maintaining parent engagement, particularly among parents needing more support with depression, stress, parenting confidence, and adolescent problem behaviors. Higher intervention engagement was beneficial for quality time and parent anxiety. This study (R01MH122213-01S1) was registered September 15th, 2020.

The state of youth mental health has been declared a public health crisis, with one in five adolescents experiencing anxiety or depression and reporting that their mental healthcare needs were not addressed in the past year (Office of the Surgeon General, OSG, 2021). Parents are critical sources of support for their children’s mental health, particularly during early adolescence (Wang et al., 2019). Specifically, consistent family management practices and healthy parenting relationships protect youth from developing later problem behaviors despite other risk factors (e.g., poverty; Stormshak et al., 2011). Moreover, there is robust evidence that behavioral parenting interventions can improve youth mental health during this critical developmental window (Stormshak et al., 2011; Yap et al., 2019). Behavioral parenting interventions based on social learning theory improve child behavior and parent–child relationships by increasing positive parenting practices (e.g., praise, quality time) and reducing coercive parent–child interactions (Patterson, 1982; Sitnick et al., 2015). However, there are significant barriers that limit parents’ access to traditional evidence-based behavioral parenting interventions, including long waiting lists for providers, service expenses, and scheduling difficulties (Castro-Ramirez et al., 2021). Further, behavioral parenting interventions often require several sessions, which contributes to low participation, high attrition, and other implementation challenges (Tully & Hunt, 2016). Therefore, there is a critical need for effective, accessible, and flexible evidence-based interventions for families to address the adolescent mental health crisis. Digital or telehealth parenting programs may be particularly beneficial for reducing barriers to accessing support for families (Hall & Bierman, 2015). However, little is known about *how* parents engage in different components of digital parenting programs, which may include both asynchronous access to evidence-based content on child behavior and parenting skills as well as telehealth clinical support. The current study seeks to investigate patterns of parent engagement in a digital parenting program, the Family Check-Up (FCU) Online, and relationships between patterns of engagement, family contextual factors, and child and parenting outcomes.

## The Effectiveness of Online Parenting Interventions with and without Clinical Support

There is a growing number of online parenting programs that include both online components and access to telehealth clinical support (e.g., licensed therapist, context expert), with rigorous studies on some of these programs supporting their efficacy in reducing children’s emotional and behavior problems. A meta-analysis that included 28 studies of 16 unique online parenting programs found significant effects on reducing child problem behaviors and anxiety, and on improving parenting skills (Spencer et al., 2020). Importantly, authors found that the strength of effect sizes did not differ across programs that did or did not offer clinical support. However, other research suggests that including clinical support in digital parenting programs increases parent engagement (Hall & Bierman, 2015) and bolsters program effectiveness for key intervention outcomes (e.g., parent stress; McAloon & Armstrong, 2024). Limitations of existing reviews and meta-analyses of online parenting interventions include a lack of specificity regarding the content of online parenting interventions and inattention to important demographic features of the samples (e.g., race, ethnicity, family income).

## Measuring Engagement in Parenting Interventions Delivered In-Person

Although research on parent engagement in online interventions is in its early stages, there is a substantial body of work on parents’ participation in in-person evidence-based parenting interventions delivered in clinical or home-based settings. These interventions are largely delivered with a high degree of structure and recommended curriculum (e.g., weekly or bi-weekly), and in an individual or group format (e.g., Webster-Stratton & Reid, 2018). Although it’s clear that experiencing benefits of parenting interventions is contingent on parent engagement in the intervention, authors often do not document rates of engagement (Chacko et al., 2016). In the literature on traditional, in-person parenting interventions, intervention engagement is often defined by ongoing attendance (Ingoldsby, 2010). A review of the literature on parental engagement proposes the CAPE conceptual model, which consists of recruitment/enrollment (Connect), retention (Attend), involvement (Participate), and implementation of newly learned strategies and techniques (Enact; Piotrowska et al., 2017). Consistent with this model, research generally indicates that both session attendance and out-of-session engagement with intervention content (e.g., completion of home practice assignments to support implementation of newly learned strategies) are positively associated with intervention outcomes (Berkel et al., 2011). Specifically, intervention attendance has been found to predict positive changes in parenting behaviors (Brody et al., 2006). However, there is also evidence that while some degree of attendance is necessary to achieve intervention outcomes, it may not be *sufficient* for some families to achieve change (Staudt, 2007). For example, in a study of parents participating in group-based parent training, researchers found that quality of participation (e.g., homework completion) was a stronger predictor of changes in parenting and parenting self-efficacy than attendance (Clarke et al., 2015). Understanding intervention engagement may therefore be particularly important to recognize outcomes, especially considering how group-based parenting interventions with prescribed curriculums and number of sessions may have limited opportunities to tailor programming to fit families’ unique goals, needs, and contextual factors.

## Engagement in Online Versus In-Person Parenting Interventions

Although online interventions are hypothesized to reduce barriers to access and engagement in parenting support programs, little research has explored how parents engage in digital parenting interventions, particularly those with dual components (i.e., asynchronous content and telehealth coaching). Some research suggests that digitally delivered parenting programs may be associated with higher completion rates compared with in-person program delivery (Breitenstein et al., 2014). However, in a study of the free, evidence-based and self-directed online program, Parentworks, over half of parents who enrolled in the program did not start it, and only 8% completed the full program (Dadds et al., 2019).

Research suggests that families’ contextual risk factors may affect their engagement in parenting interventions. For example, there is some evidence suggesting that high levels of parenting or family stress may function as a barrier *or* as a facilitator to in-person parenting intervention engagement (e.g., Williamson et al., 2016)*.* Low socioeconomic status and economic strain are generally associated with reduced engagement in parenting interventions (Berry et al., 2023). Research on parents’ engagement in the Family Check-Up program, a brief, individualized, evidence-based preventive parenting intervention based on motivational interviewing principles (Dishion & Stormshak, 2007), has found that parents with high levels of caregiving and family stress demonstrate *more* engagement (e.g., attendance, amount of intervention time) than parents with lower stress levels (Connell et al., 2007; Smith et al., 2018), suggesting that parents appropriately “self-select” into the services they need.

Research evaluating the influence of family demographic and contextual risk factors on digital intervention engagement has been much more limited. One recent study focused on U.S. military families with children ages 5–12 found that both household income and fathers’ depressive symptoms were negatively associated with engagement (i.e., attendance, enrollment) in a self-directed, online parenting program (Cai et al., 2024). Importantly, in comparison to a parenting program delivered in an in-person, group format, Cai and colleagues (2024) found that the self-directed online delivery was associated with higher enrollment and attendance. More research is needed to understand whether these findings are generalizable to other, non-military families. Another recently published study compared traditional office-based parent–child interaction therapy (PCIT) with internet-delivered PCIT (iPCIT) among families of 3–5-year-old children (Sanchez et al., 2024). Sanchez and colleagues (2024) found that iPCIT improved attendance rates, especially for families from minoritized racial/ethnic backgrounds. They also found that among families with high parent stress, office-based PCIT was associated with lower dropout rates and improved treatment alliance and satisfaction compared to the online model (Sanchez et al., 2024). Overall, there is some evidence that digital intervention engagement may vary based on family demographic and contextual factors, but the research is limited.

## Online Intervention Engagement and Parenting Outcomes

Beyond understanding rates and predictors of attrition, there has been limited research focused on investigating patterns of engagement in online parenting programs, and whether engagement is linked to intervention outcomes. Some prior work has found that the number of self-directed online sessions completed was positively associated with reductions in child problem behavior in a study of Triple P Online (Dittman et al., 2014); however, information on engagement is scarce. For online parenting interventions involving both online content and telehealth parenting support, it would be especially important to understand patterns of engagement with each component. Understanding these patterns could shed light on how engagement with each component is differentially associated with contextual risk factors as well as treatment outcomes. Therefore, identifying patterns of engagement in digital parenting interventions, particularly those with multiple components, can be critical to understanding intervention effects.

## The Family Check-Up (FCU) Online for Early Adolescents in Middle School

The Family Check-Up Online (FCU Online) is a flexible parenting intervention involving an app and supplemental family coaching sessions. The FCU Online was originally developed to reduce problem behavior in early adolescence and includes online materials incorporating parent assessments with feedback (Stormshak et al., 2019). The FCU Online was adapted in response to the COVID-19 pandemic to include content focused on supporting families with pandemic-related stressors such as online schooling, isolation, adaptive parenting, mental health problems, and coping with stress (Connell & Stormshak, 2023; Mauricio et al., 2024). It includes five modules on effective parenting that were adapted from the Everyday Parenting Curriculum (Dishion et al., 2012), along with supplemental telehealth coaching that supports parents in making behavioral changes in their parenting and family processes, such as conflict (Stormshak et al., 2019). Initial results of the intervention demonstrated significant improvements in parent anxiety, depression, perceived stress, parenting skills, and family functioning among parents of early adolescent middle schoolers ages 10–14 (Connell & Stormshak, 2023). However, to date, no study has yet explored patterns of parent engagement in the FCU Online and the relation between patterns of engagement, contextual factors, and outcomes.

## Current Study

This exploratory study sought to identify rates and types of parental engagement in the FCU Online to understand whether differences in engagement were related to parent contextual factors or differential outcomes. The specific research questions investigated were: (1) What are the rates of parent intervention engagement in the FCU Online coaching and the app? (2) Did parent intervention engagement differ based on individual or contextual factors (i.e., race/ethnicity, SES, parenting confidence, anxiety, depression, stress, adolescent age, and problem behaviors)? and (3) Does engagement level in the FCU Online predict differential outcomes for parents and their families six months after intervention enrollment? Our first research question was exploratory, as the evidence around digital intervention engagement is limited. For our second and third questions, we hypothesized (2) that parents with greater individual and contextual risk factors would have higher rates of engagement and demonstrate greater improved outcomes, consistent with prior findings of the FCU Online (Connell & Stormshak, 2023; Hails et al., 2025a; Stormshak et al., 2019); and (3) that greater engagement in the FCU Online intervention would predict improved parenting skills and confidence, improved parent mental health, and fewer adolescent problem behaviors.

## Method

### Participants

Participants in the original study included 161 parents (i.e., any primary caregiver including biological or adoptive parents, grandparents, etc.) and their early adolescent in middle school (i.e., ages 10–14). The current study used baseline and 6-month post-enrollment data from parents who were randomized to the intervention group (*N* = 74). Parent participants in the current study were on average 41.91 years old (*SD* = 5.99). Parents were primarily female (94.6%, *n* = 70), and were White (83.8%, *n* = 62), Hispanic/Latinx (18.9%, *n* = 14), bi/multiracial (13.5%, *n* = 10), Asian (1.4%, *n* = 1), and Black (1.4%, *n* = 1). Parents were mostly employed full time (59.5%), and most had a 4-year college degree or higher (60.8%, *n* = 45), 2 years of Junior college/Associate’s degree (13.5%, *n* = 10), or partial college/specialized training (13.5%, *n* = 10). Regarding income, 23.0% of families reported an income of 29,999 USD or under. Early adolescent participants were 48.6% female and 47.3% male (with 4.1% indicating “other” gender) and on average 12.61 years old (*SD* = 12.53 months). Early adolescents were White (73.0%, *n* = 54), Hispanic/Latinx (25.7%, *n* = 19), bi/multiracial (24.3%, *n* = 18), and Black (2.7%, *n* = 2). This sample of families was racially and ethnically representative of the demographics of the Pacific Northwest in the U.S. (85.6% White, 14.9% Hispanic/Latinx; U.S. Census Bureau, 2024).

### Procedures

Data for the current study included participants from an original effectiveness trial of the FCU Online model (Connell & Stormshak, 2023). Parents were recruited via flyers, social media, community agencies, pediatric settings, and mail. Interested parents then contacted the study team. A study team member explained the study to parents, assessed eligibility and confirmed the parent met eligibility criteria, and reviewed informed consent. Parents were eligible to participate if they were the legal guardian of a child ages 10 to 14, spoke English or Spanish (because the FCU Online was only available in these languages), had an email and a smart phone with text-messaging, and endorsed significant levels of depression on the 2-item Patient Health questionnaire (PHQ; Kroenke & Spitzer, 2001) and/or significant stress associated with the COVID-19 pandemic using the 4-item Perceived Stress Scale (PSS; Cohen et al., 1994) on their initial screening. Only one parent per household or family was eligible to participate.

Parents completed a baseline assessment and follow-up assessments 2, 4, and 6 months later. Assessments included questions about family demographics, parenting skills, parent mental health, and child behaviors. Parents completed assessments online or via phone call based on their preference. Families were then randomly assigned to the FCU Online intervention condition or a waitlist control. Families were paid a total of $300 to participate. Data can be made available upon request to the fifth author; this study was pre-registered on ClinicalTrials.gov. All procedures were approved by the IRB.

FCU Online modules focused on the following content, listed in chronological order: Healthy Behaviors for Stressful Times, Positive Parenting, Rules & Consequences, Support School Success, and Communication. Each week, the next module unlocked for parents to access. Modules were developed to support parenting concerns common among parents of early adolescents and include developmentally appropriate information and videos. Each module took approximately 15–20 min to complete (75–100 min total), and content was provided in short intervals. Parents were able to start where they left off after exiting the app and could re-watch the content at any time. Any time spent re-watching modules was counted in the parent’s total time on the app. Parents randomly assigned to FCU Online were able to meet one-on-one with a family coach via telehealth (i.e., HIPAA compliant video conferencing, phone calls, texts). Family coaches were master’s or doctoral-level clinicians trained in the FCU Online. The family coach enrolled the parent in the FCU Online app during a brief phone or video call and conducted an initial interview to learn about the family and to set intervention goals. Parents were then invited to participate in follow-up telehealth family coaching sessions. The basic model of the FCU Online was roughly 5–6 phone sessions of about 20–30 min (around 120 total minutes) for each parent. However, intervention dosage was highly flexible, and parents were invited to participate in as many coaching sessions as they desired during the year they had access to the app, depending on parent needs and preference. Coaching session content included a general check-in, reviewing parent progress in the app, discussing and practicing skills parents learned in the app while linking practice to established goals, troubleshooting barriers to using skills, and developing a plan for the parent’s next steps using the app and applying the skills at home before the subsequent session. Coaching session topics were based on parent preference and typically addressed the most recent topic the parent had covered in the app. Sessions allowed an opportunity for parents to discuss individual and contextual challenges and to tailor the online intervention more specifically to their context. Parents were additionally provided referrals and resources for services needed (e.g., therapists, housing support). Prior studies with different samples of early adolescents in middle school have found the FCU Online resulted in improved parenting confidence when compared to control groups, as well as reduced emotional problems for children, with at-risk youth demonstrating stronger effects compared to those with minimal risk (Connell & Stormshak, 2023).

### Measures

#### Demographic Characteristics

Parents completed a demographics questionnaire that asked about parent and child race/ethnicity, sex, and age, as well as parent income and education.

#### FCU Online Coaching Data

Family coaches completed the Parent Consulting Log (PACL; Winter & Dishion, 2007) after each coaching session. Information extracted from the PACL included the total number of minutes engaged in coaching sessions, total number of coaching sessions, and which modules and content were discussed in coaching sessions.

#### FCU Online App Usage Data

Information on app engagement was collected automatically by the app. We extracted these data, including total number of times the app was visited, total minutes spent on the app overall, and total number of minutes spent in each module.

#### Parenting Skills

Parents completed an adapted version of the Parenting Young Children scale (PARYC; McEachern et al., 2012), a 21-item self-report measure of parenting skills on a 5-point Likert type scale ranging from 0 = *Never* to 4 = *Very Often*. The measure includes four subscales, including Quality Time (5 items, α = .74, e.g., “In the past month, did you do an enjoyable activity together?”), Positive Parenting (2 items, α = .56, e.g., “In the past month, did you notice and praise your child's good behavior?”), Proactive Parenting (7 items, α = .79, e.g., “In the past month, did you break tasks into small steps e.g., Please make your bed and pick up your dirty laundry, rather than Clean up your room?”), and Limit Setting (7 items, α = .76, e.g., “In the past month, did you stick to your rules and not change your mind?”). Scores were derived by calculating the mean across items.

#### Parenting Confidence

Parents completed the Parenting Competence scale (Prevention Science Institute, 2016), a 28-item measure assessing parents’ confidence using specific parenting skills on a scale ranging from 1 = *Not at All Confident* to 5 = *Very Confident* (α = .87, e.g., “How confident do you feel providing praise and encouragement for good behavior?” and “How confident do you feel monitoring school work?”). Scores were derived by calculating the mean score across items.

#### Parent Anxiety

Parents completed the Generalized Anxiety Disorder-2 (GAD-2; Kroenke et al., 2007), a 2-item measure of anxiety in the last month on a 4-point Likert type scale ranging from 0 = *Not at All* to 3 = *Nearly Every Day* (α = .84, i.e., “How many days in the last month were you feeling nervous, anxious, or on edge?” and “How many days in the last month were you not being able to stop or control your worrying?”). Scores were derived by calculating the mean across items.

#### Parent Depression

Parents completed the Patient Health Questionnaire-9 (PHQ-9; Kroenke & Spitzer, 2001), a screening measure to assess for depressive symptoms in the last two weeks. Parents completed 9 items on a 4-point Likert type scale ranging from 0 = *Not at All* to 3 = *Nearly Every Day* (α = .86, e.g., “In the last two weeks, how often have you been bothered by feeling down, depressed, or hopeless?” and “In the last 2 weeks, how often have you been bothered by feeling bad about yourself, or that you are a failure, or have let yourself or your family down?”). Scores were derived by calculating the mean score across items. For screening purposes, a 2-item version of the measure was used. Eligibility included a highly sensitive cutoff score of 1 or above (experiencing symptoms for several days; Connell & Stormshak, 2023).

#### Parent Stress

Parents completed the Perceived Stress Scale (PSS; Cohen et al., 1994), a 14-item measure of stress in the last month on a 5-point Likert type scale ranging from 0 = *Not at All* to 4 = *Very Often* (α = .90, e.g., “In the last month, you have felt that you were unable to control the important things in your life?” and “In the last month, you have felt that you were on top of things?”). Scores were derived by calculating the mean score across items. For screening purposes, a 4-item version of the measure was used, and eligibility included a cutoff score of 2 or above on any item (sometimes; Connell & Stormshak, 2023).

#### Adolescent Problem Behaviors

Parents completed the Strengths and Difficulties Questionnaire (SDQ; Goodman, 1997), a 25-item measure about adolescent emotional and behavioral problems. Responses were on a 3-point Likert type scale ranging from 0 = *Not True* to 3 = *Certainly True* (α = .85, e.g., “My child is restless, overactive, cannot stay still for long”). Items were summed for a total score; the total score was used for this study.

### Data Analytic Plan

Analyses were conducted utilizing the Statistical Package for Social Sciences (SPSS) software version 28 (IBM Corp, 2023). Missing data were handled via pairwise deletions. Coaching and app engagement were analyzed separately as it was possible for a parent to engage in both the app and coaching, the app only, or coaching only. To assess the first research question related to rates of parent engagement in the app, we used frequencies to determine the number of minutes spent in coaching sessions or using the app. We classified parents into one of two a priori-defined categories of engagement: Typical Engagement and High Engagement. For coaching, the cut point between typical and high levels of engagement was 160 min, determined by (1) the standard amount of time for the FCU Online (i.e., 5–6 phone sessions each 20–30 min, around 120 min total) while conservatively including more intervention time due to the flexibility of intervention dosage, and (2) the mean number of minutes engaged in coaching (160 min). For the app, the engagement cut point was the mean level of engagement at 136 min.

For research question two, we used One-Way between subjects’ Analysis of Variance (ANOVAs) to evaluate whether parent engagement in the FCU Online app or coaching differed based on individual (e.g., race/ethnicity, education, confidence, anxiety, depression, stress) or contextual factors (e.g., income, adolescent age, adolescent problem behaviors at baseline). Finally, research question three used a Multivariate Analysis of Covariance (MANCOVA) to determine whether parent engagement in coaching sessions and the app was related to differential outcomes for parents and their children (i.e., parenting skills and confidence, parent mental health, and adolescent problem behaviors) six months after intervention enrollment, after controlling for baseline characteristics (i.e., parenting skills and confidence, parent mental health, and adolescent problem behaviors) and income. Income was included as a covariate in all analyses as low SES has been linked with lower intervention engagement (Berry et al., 2023). For both analyses, effect sizes with *n*^*2*^ (eta squared) were used as this is recommended for interpretation of these analyses (Henson, 2006).

## Results

Frequency counts revealed that all 74 parents assigned to the intervention condition engaged in the FCU Online. One parent engaged in coaching only, another parent only engaged in the app, and all other parents engaged in both coaching and the app. Parents were divided into two groups based on their rate of engagement (Typical Engagement, High Engagement) in coaching sessions and the app. For coaching, the Typical Engagement group included parents that completed up to 160 min of coaching; the High Engagement group included parents with above average coaching time (161 min or more). Parents primarily engaged in coaching sessions via phone call or text. For the app, Typical Engagement was determined by the mean number of minutes using the app (*M* = 136 min) or less. Overall, for parents who engaged in coaching sessions (question 1), 56.8% of parents were in the Typical Engagement group (*n* = 42) and 43.2% were in the High Engagement group (*n* = 32). For the app, 52.7% of parents were in the Typical Engagement group (*n* = 39) and 47.3% were in the High Engagement group (*n* = 35).

Table 1 shows the descriptive statistics of the distributions of family characteristics in each engagement level in coaching and the app. Table 2 shows results of analyses examining whether parent intervention engagement differed based on individual or contextual factors (hypothesis 2). Parents in the High Engagement group for coaching had higher levels of depression (*F*(1) = 4.59, *p* < .05, *n*^*2*^ = .060) and perceived stress (*F*(1) = 5.06, *p* < .05, *n*^*2.*^ = .067), lower parenting confidence (*F*(1) = 5.70, *p* < .05, *n*^*2.*^ = .073), and had adolescents with higher problem behaviors (*F*(1) = 6.46, *p* < .05, *n*^*2.*^ = .082) compared to the Typical coaching group, all with medium effect sizes. No other significant differences were found in coaching engagement for parent race, ethnicity, education, income, anxiety, or age of the adolescent. Further, no significant differences were found in parent engagement in the app based on individual or contextual factors.

**Table 1:** Descriptive statistics on distributions of family characteristics across coaching and app engagement levels

	Coaching Engagement		App Engagement	
	Typical Engagement (*N* = 42)	High Engagement (*N* = 32)	Typical Engagement (*N* = 39)	High Engagement (*N* = 35)
	*M (SD) or % endorsed*	*M (SD) or % endorsed*	*M (SD) or % endorsed*	*M (SD) or % endorsed*
Race (White)	81.0%	87.5%	82.1%	85.7%
Ethnicity (Latino)	16.7%	21.9%	12.8%	25.7%
Teen age (months)	150.98 (12.46)	151.63 (12.81)	151.00 (12.96)	151.60 (12.21)
Education (≥ College)	64.3%	56.2%	64.1%	57.2%
Income (≤ $29,999)	11.9%	37.5%	20.5%	25.7%
Parenting confidence	4.03 (.49)	3.73 (.62)	3.91 (.47)	3.89 (.66)
Anxiety	1.00 (.80)	1.42 (1.04)	1.15 (.89)	1.21 (.98)
Depression	0.62 (.52)	0.92 (.69)	0.72 (.63)	0.78 (.61)
Perceived stress	1.69 (.52)	2.01 (.69)	1.86 (.64)	1.80 (.59)
Adolescent problem behaviors	12.38 (6.57)	16.72 (8.10)	13.39 (6.83)	15.23 (8.25)

All variables about parents unless otherwise stated. Education: Percent of parents with a standard 4-year university or college education or more. Income: Parents making $29,999 or less consistent with federal poverty line threshold in 2020 (ASPE, 2020)

**Table 2:** One-way analyses of variance on whether parent engagement in coaching sessions and the app differed based on individual and contextual factors

	*SS*	*df*	*MS*	*F*	*p*	*n*^*2*^	*95% CI* [LL, UL]
Coaching Engagement							
Parent race	0.701	1	0.701	0.119	0.731	0.002	[0.000, 0.063]
Parent ethnicity (y/n Hispanic)	0.049	1	0.049	0.314	0.577	0.004	[0.000, 0.078]
Adolescent age (months)	7.645	1	7.645	0.048	0.827	0.001	[0.000, 0.050]
Education	0.284	1	0.284	0.119	0.731	0.002	[0.000, 0.063]
Income	38.311	1	38.311	2.410	0.125	0.032	[0.000, 0.144]
Parenting confidence	1.699	1	1.699	5.699	0.020*	0.073	[0.001, 0.206]
GAD-Parent anxiety	3.232	1	3.232	3.908	0.052	0.051	[0.000, 0.175]
PHQ-9-Parent depression	1.656	1	1.656	4.587	0.036*	0.060	[0.000, 0.187]
PSS-Parent perceived stress	1.831	1	1.831	5.062	0.028*	0.067	[0.000, 0.197]
SDQ-Total problem behaviors	341.748	1	341.748	6.464	0.013*	0.082	[0.003, 0.218]
App Engagement							
Parent race	6.008	1	6.008	1.037	0.312	0.014	[0.000, 0.107]
Parent ethnicity (y/n Hispanic)	0.307	1	0.307	1.999	0.162	0.027	[0.000, 0.134]
Teen age (months)	7.824	1	7.824	0.049	0.825	0.001	[0.000, 0.051]
Education	2.367	1	2.367	1.003	0.320	0.014	[0.000, 0.106]
Income	21.364	1	21.364	1.324	0.254	0.018	[0.000, 0.116]
Parenting confidence	0.008	1	0.008	0.026	0.872	0.000	[0.000, 0.043]
GAD-Parent anxiety	0.067	1	0.067	0.077	0.782	0.001	[0.000, 0.057]
PHQ-9-Parent depression	0.067	1	0.067	0.176	0.676	0.002	[0.000, 0.068]
PSS-Parent stress	0.071	1	0.071	0.183	0.670	0.003	[0.000, 0.070]
SDQ-Total problem behaviors	62.719	1	62.719	1.105	0.297	0.015	[0.000, 0.109]

*p < 0.05

*LL* Lower Level, *UL* Upper Level, *PARYC* Parenting Young Children, *GAD* Generalized Anxiety Disorder, *PHQ* Patient Health Questionnaire, *PSS* Perceived Stress Scale, *SDQ* Strengths and Difficulties Questionnaire

Significant values are bolded

Table 3 demonstrates whether parent engagement in the FCU Online coaching sessions and app was related to differential outcomes in parenting skills, parent mental health, and adolescent problem behaviors at six months post-test. Engagement level predicted quality time, such that parents with more engagement in coaching sessions reported improved quality time with their adolescent at six-months post-test, controlling for baseline scores on quality time, with a medium effect size (*F*(1) = 8.30, *p* < .05, *n*^*2*^ = .135; hypothesis 3). Findings also indicated that parents with more engagement in the app reported reduced anxiety at six-months post-test, controlling for baseline scores on anxiety, with a medium effect size (*F*(1) = 4.15, *p* < .05, *n*^*2.*^ = .073). Engagement level in either coaching or the app did not predict any other outcomes at six months, including the parenting skills of positive parenting, proactive parenting, limit setting, parenting confidence, parent depression or stress, or adolescent problem behaviors.

**Table 3:** Multivariate Analysis of Covariance on Parent Engagement in Coaching Sessions and App on Parenting Skills, Parent Mental Health, and Child Behavioral Problems at 6 Months

	*M*	*SD*	*SS*	*df*	*MS*	*F*	*p*	*n*^*2*^	*95% CI* [LL, UL]
Coaching Engagement									
PARYC-Quality time	2.65	0.67	1.639	1	1.639	8.300	0.006**	0.135	[0.110, .614]
PARYC-Positive parenting	2.96	0.70	0.162	1	0.162	0.487	0.488	0.009	[− 0.213, .441]
PARYC-Proactive parenting	2.91	0.63	0.024	1	0.024	0.095	0.759	0.002	[− 0.326, .239]
PARYC-Limit setting	3.00	0.56	0.542	1	0.542	2.763	0.102	0.050	[− 0.459, .043]
Parenting Confidence	4.01	0.64	0.023	1	0.023	0.103	0.749	0.002	[− 0.226, 0.312]
GAD-Parent anxiety	0.91	0.83	0.540	1	0.540	1.458	0.233	0.027	[− 0.553, 0.137]
PHQ-9-Parent depression	0.56	0.62	0.138	1	0.138	0.723	0.399	0.013	[− 0.353, 0.143]
PSS-Parent stress	1.54	0.67	0.701	1	0.701	3.719	0.059	0.066	[− 0.483, 0.009]
SDQ-Total problem behaviors	11.82	7.12	29.816	1	29.816	1.617	0.209	0.030	[− 3.978, 0.891]
App Engagement									
PARYC-Quality time	2.65	0.67	0.057	1	0.057	0.251	0.618	0.005	[− 0.191, 0.318]
PARYC-Positive parenting	2.96	0.70	0.013	1	0.013	0.039	0.844	0.001	[− 0.339, 0.279]
PARYC-Proactive parenting	2.91	0.63	0.190	1	0.190	0.775	0.383	0.014	[− 0.380, 0.148]
PARYC-Limit setting	3.00	0.56	0.003	1	0.003	0.016	0.898	0.000	[− 0.227, 0.258]
Parenting Confidence	4.01	0.64	0.097	1	0.097	0.435	0.512	0.008	[− 0.169, 0.335]
GAD-Parent anxiety	0.91	0.83	1.463	1	1.463	4.145	0.047*	0.073	[− 0.638, − 0.005]
PHQ-9-Parent depression	0.56	0.63	0.103	1	0.103	0.537	0.467	0.010	[− .318, 0.148]
PSS-Parent stress	1.54	0.67	0.125	1	0.125	0.625	0.433	0.012	[− .332, 0.144]
SDQ-Total problem behaviors	11.82	7.12	3.263	1	3.263	0.172	0.680	0.003	[− 2.799, 1.839]

*n* = 65. *p < 0.05, **p < 0.01

*LL* Lower Level, *UL* Upper Level, *PARYC* Parenting Young Children, *GAD* Generalized Anxiety Disorder, *PHQ* Patient Health Questionnaire, *PSS* Perceived Stress Scale, *SDQ* Strengths and Difficulties Questionnaire

Significant values are bolded

## Discussion

Engagement in digital parenting interventions is an important yet understudied area. While session attendance and engagement with in-person intervention content has been positively associated with intervention outcomes and has predicted positive changes in parenting behaviors (Berkel et al., 2011; Brody et al., 2006), research on parent patterns of engagement in digital parenting interventions is scarce. The FCU Online has demonstrated positive impacts on parenting skills, parent mental health and wellbeing, and child and adolescent behaviors (Connell & Stormshak, 2023; Hails et al., 2025a). To date, no study has investigated patterns of parent engagement in the FCU Online, and how these levels of engagement are related to family contextual factors and intervention outcomes. To fill this gap, the current study included parents experiencing pandemic-related stress and explored (1) the rates of parent engagement in the FCU Online coaching or the app; (2) whether parent engagement differed based on individual or contextual factors; and (3) whether engagement level in the FCU Online predicted differential outcomes for parents and their families after six months.

First, all parents that were assigned to the FCU Online intervention condition engaged in the intervention. While one parent engaged in only coaching and another in only the app, all other parents engaged in both aspects of the FCU Online. More than half of parents had Typical Engagement in coaching sessions (56.8%) and the app (52.7%) respectively, while 43.2% of parents were in the High Engagement group for coaching sessions and 47.3% for the app. Considering the challenge of engaging parents in digital and in-person parenting interventions (e.g., Chacko et al., 2016; Dadds et al., 2019), these engagement levels may indicate the FCU Online was particularly successful in maintaining parent engagement. As prior work suggests the availability of clinical coaching support in digital parenting interventions can improve engagement (Hall & Bierman, 2015), it is possible that offering both an app and supplemental coaching supported parent intervention engagement. This is an important finding given that the current sample was comprised of parents of middle school students, and effective parenting programs during early adolescence are critical for youth mental health (Stormshak et al., 2011; Yap et al., 2019). Moreover, considering the barriers to accessing traditional in-person parenting programs (Castro-Ramirez et al., 2021), the high levels of engagement in the FCU Online demonstrate that digital parenting interventions may increase access and acceptability among families with early adolescents in middle school.

Engagement level was found to differ by four risk factors. Specifically, parents with more depression and perceived stress, lower parenting confidence, and who had adolescents with more problem behaviors were significantly more likely to have high engagement in coaching sessions. Results are somewhat inconsistent with prior work demonstrating associations between parent depression and low engagement in self-directed, online parenting programs (Cai et al., 2024), and links between low in-person intervention engagement and family stress (Williamson et al., 2016) and low socioeconomic status (Berry et al., 2023). The current study demonstrated the reverse, in that higher parent depression and stress were associated with higher levels of coaching engagement. As the current study only included a sample of parents experiencing heightened stress, this suggests that the coaching support in the FCU Online may have been particularly useful for parents with more distress. This is consistent with prior work on the in person FCU intervention where parents with more stress had higher engagement than parents with lower stress (Connell et al., 2007; Smith et al., 2018).

Further, among this sample of parents of early adolescents, problem behaviors and low parenting confidence were significantly related to more intervention engagement. It is possible that parents were more engaged in coaching sessions because they had greater concerns about their adolescents’ behaviors and were eager to receive support in managing those behaviors. Similarly, parents with lower parenting confidence may have been more highly engaged in coaching sessions due to a desire to receive more interpersonal parenting support and develop more skills to manage their adolescents’ behaviors. This is consistent with previous research indicating that family conflict and deviant peer involvement predicted greater engagement in the in-person, school-based version of the FCU (Connell et al., 2007), and that clinical support can increase parent engagement in digital parenting interventions (Hall & Bierman, 2015). No other contextual or individual risk factors (e.g., parent race, ethnicity, education, income, anxiety, or age of adolescent) were associated with different levels of engagement in coaching. Further, neither contextual nor individual risk was associated with different levels of engagement in the app.

Finally, we investigated whether engagement level predicted outcomes for families after six months. First, when parents had more engagement in coaching sessions, this predicted improved quality time with their early adolescent. This is consistent with prior work demonstrating that behavioral parenting interventions can improve parent–child relationships by improving parenting practices such as quality time (Patterson, 1982; Sitnick et al., 2015). Second, parents with higher engagement in the app had decreased anxiety at 6 months post-test, after controlling for baseline anxiety. Prior work on the FCU Online has shown similar positive impacts on parent anxiety (Connell & Stormshak, 2023). As parents in the current sample were experiencing high levels of pandemic-related stress, the improvements in parent anxiety by simply engaging in a digital parenting skills app is promising. Further, while not statistically significant, parents with more anxiety appeared to be in the High Engagement group more than the Typical Engagement group (*M* = 1.42 and *M* = 1.00, respectively, *p* = .052). Overall, higher engagement in the FCU Online had some positive results for families experiencing many contextual stressors. Finally, no other differences in parenting skills or confidence, parent depression or stress, or adolescent behavioral outcomes were found based on parent coaching or app engagement level.

### Implications for Prevention Practice

The FCU Online for early adolescents in middle school was adapted in response to the COVID-19 pandemic and was specifically designed to target challenges for parents experiencing stress. Parents provide critical support to their early adolescent’s mental health (Wang et al., 2019). As behavioral parenting interventions can support early adolescent mental health (Stormshak et al., 2011; Yap et al., 2019), and as the FCU Online predicted improved quality time and decreased parent anxiety, the program has the potential to provide parents with necessary tools to minimize the current youth mental health crisis. Notably, all parents assigned to the FCU Online intervention engaged in the program. The high level of engagement in the program, particularly among parents with more depression and stress, lower parenting confidence and with adolescents engaging in more problem behaviors, indicated high acceptability of the program among this sample. As there are widespread barriers to accessing evidence-based parenting interventions (Castro-Ramirez et al., 2021), the high engagement in the FCU Online indicates a promising solution for improving access to critical family services.

### Limitations and Future Directions

The current study included a sample of 74 parents of early adolescents in middle school. Future research should include a larger sample size of parents to better understand variations in engagement patterns among parents in this digital parenting intervention. Further, participants were primarily female and White, thus limiting the generalizability of findings. Future work should include a more diverse sample (e.g., racially/ethnically, more male caregivers) to gain a better understanding of engagement across groups. Challenges with engaging fathers in parenting interventions is widespread (Gonzalez et al., 2023), and future work should include a focus on enhancing father engagement in digital parenting programs. Additionally, as coach characteristics such as cultural competency or quality of feedback may influence parent engagement and outcomes, future work should investigate coach and coaching session characteristics as they were not measured in the current study (e.g., Hails et al., 2025b). This study measured parent engagement in the app; however, information such as motivation to participate in the intervention and program satisfaction were not collected. Future work should assess how motivation and satisfaction are associated with levels of engagement. This study focused specifically on parents with early adolescent children. Future work should investigate parent patterns of intervention engagement among different age groups, including younger children, to determine potentially differing engagement patterns, risk factors, and links to outcomes.

## Conclusion

The FCU Online is flexible, designed to support parents in ways they identify as being most beneficial, and has demonstrated multiple positive outcomes for families of early adolescents (Connell & Stormshak, 2023). Providing parents with agency in the amount and type of intervention engagement they wanted may have been highly beneficial for families in the wake of COVID-19. As rates of engagement were high among families in this intervention, the FCU Online demonstrated acceptability from this sample of highly stressed families of early adolescents in middle school. Exploring patterns of parent engagement can ultimately support our understanding of how flexible and accessible digital parenting interventions, such as the FCU Online, can empower parents and promote their ability to engage in changes to their parenting and their families in ways they desire.

## Data Availability

Data can be made available upon request to Elizabeth A. Stormshak; the original study was pre-registered on ClinicalTrials.gov (NCT03060291).
